# Rumen-protected conjugated linoleic acid supplementation to dairy cows in late pregnancy and early lactation: effects on milk composition, milk yield, blood metabolites and gene expression in liver

**DOI:** 10.1186/1751-0147-52-16

**Published:** 2010-02-18

**Authors:** Tanja Sigl, Gregor Schlamberger, Hermine Kienberger, Steffi Wiedemann, Heinrich HD Meyer, Martin Kaske

**Affiliations:** 1 Physiology Weihenstephan, Technische Universitaet Muenchen, Weihenstephaner Berg 3, Freising-Weihenstephan, Germany; 2 Bioanalytik, Technische Universitaet Muenchen, Versuchsstation Thalhausen, Freising-Weihenstephan, Germany; 3 Clinic for Cattle, University of Veterinary Medicine, Bischofsholer Damm 15, Hannover, Germany

## Abstract

**Background:**

Conjugated linoleic acid (CLA) is a collective term for isomers of octadecadienoic acid with conjugated double-bond system. Thus, it was the objective to investigate whether milk composition and metabolic key parameters are affected by adding CLA to the diet of dairy cows in the first four weeks of lactation.

**Methods:**

A study was carried out with five primiparous cows fed a CLA supplemented diet compared to five primiparous cows without CLA supplementation. CLA supplemented cows received 7.5 g CLA/day (i.e. 50% *cis*(c)9,*trans*(t)11- and 50% *t*10,*c*12-CLA) starting two weeks before expected calving and 20 g CLA/day (i.e. 50% *c*9,*t*11- and 50% *t*10,*c*12-CLA) throughout day 1 to 28 of lactation.

**Results:**

The CLA supplement was insufficiently accepted by the animals: only 61.5% of the intended amount was ingested. Fed CLA were detectable in milk fat, whereas contents of *c*9,*t*11-CLA and *t*10,*c*12-CLA in milk fat were higher for CLA supplemented cows compared to the control group. On average over the entire treatment period, there was a decrease of saturated fatty acids (FA) in milk fat of CLA supplemented cows, combined with a higher content of monounsaturated and *trans *FA.

Our study revealed no significant effects of *c*9,*t*11- and *t*10,*c*12-CLA supplementation either on milk yield and composition or on metabolic key parameters in blood. Furthermore the experiment did not indicate significant effects of *c*9,*t*11- and *t*10,*c*12-CLA-supplementation on gene expression of peroxisome proliferator-activated receptor-alpha (PPARα), PPARγ, sterol regulatory element-binding protein-1 and tumor necrosis factor-alpha in liver tissue.

**Conclusions:**

Feeding *c*9,*t*11- and *t*10,*c*12-CLA during the first weeks after calving did not affect metabolic key parameters of blood serum or milk composition of fresh cows. Milk fatty acid composition was changed by feeding *c*9,*t*11- and *t*10,*c*12-CLA resulting in higher contents of these isomers in milk fat. High contents of long chain FA in milk fat indicate that CLA supplementation during the first four weeks of lactation did not affect massive peripheral lipomobilization.

## Background

Within the European Union, a system with fixed milk quotas per farm is applied, aiming to control total milk production and avoid surplus of milk. The quota is expressed as kg milk with a defined milk fat content, which allows the farmer to market a higher milk volume if milk fat content is low. Therefore, the possibilities to decrease milk fat content have gained interest from farmers. Furthermore, the dairy industry tries to enhance consumers trust in dairy fat by increasing the conjugated linoleic acid (CLA) content. Finally, the interest in CLA in research today is to a large extent driven by a general interest in the mechanisms of milk fat synthesis.

Extensive basic research, established predominantly by the group of Bauman and colleagues in Cornell, has shown that *trans*(t)10,*cis*(c)12-CLA reduces milk fat synthesis in the alveolar epithelial cells of the mammary gland [1] and, consequently, milk fat percentage.

Moreover, a reduction of milk fat production was considered as an option to reduce the metabolic imbalance of transition cows. The transition period, i.e. 21 days before calving until day 21 after calving [2], is a critical time frame in which the animals have to cope an enormous metabolic challenge due to the rapid onset of high milk production and a reduced dry matter intake. Managing and feeding the transition cow affects health and productivity during lactation and is the basis for economical and sustainable milk production. To meet energy requirements at the beginning of lactation, an increase of energy density of the diet by adding rumen-protected fat may be assumed. Furthermore, the energy deficit also could be reduced if the fat content of milk is lowered by a feed supplement.

It is hypothesized that a reduction of milk fat percentage may improve the metabolic resilience of high yielding dairy cows in early lactation. Thus, it was the objective to investigate whether milk composition and metabolic key parameters are affected by adding rumen-protected CLA to the diet of dairy cows in the first four weeks of lactation.

## Materials and methods

### Animals, treatments, and sampling

The study was performed according to strict federal and international guidelines on animal experimentation. The experiment was set up according to the requirements of the Bavarian State animal welfare committee. Ten primiparous Brown Swiss cows were allocated to one of two groups before calving. Cows of control group (n = 5) were fed a lactational diet with concentrates according to milk yield at the onset of lactation, cows of treatment group (n = 5) were fed the same lactational diet and concentrates. However additional CLA supplement was fed during the first four weeks of lactation to cows of the CLA-group. Group arrangement was based on how well cows accepted the fat supplement. Cows that consumed the fat concentrate during five successive test days before milk stasis (56 days before expected calving date) immediately after submission were arranged to CLA-treatment group. Due to the well known feed intake depression at the beginning of lactation, especially related to the mealy and powdery supplement, a good acceptance of the supplement by each cow of treatment group was required. The study was performed with primiparous cows exclusively to avoid lactation number as a confounding factor and due to the fact that the overall effects of CLA on milk synthesis do not depend on the lactation number [3]. All cows were housed in free stall barns fitted with rubber mats and fed the same basal diet (corn silage 43%, grass silage 25%, hay 32%; NE_L _1.36 Mcal/kg, Table 1) during the prepartum period, and then switched to a lactational diet (corn silage 60%, grass silage 23%, concentrates 12%, hay 4%, and minerals 1% of wet weight; NE_L _1.55 Mcal/kg, Table 1) immediately after parturition. The basal diet, formulated on the basis of a milk yield of 22 kg/day, was delivered once daily (0700 h) and intended to provide *ad libitum *intake (> 5% residual feed). If daily milk yield exceeded 22 kg additional concentrates were fed (0.5 kg concentrates per kg milk). Dry matter intake could not be assessed. Water was available at all times. Five cows were additionally fed a special supplement including rumen-protected CLA (encapsulation technology: spray freezed; BEWITAL GmbH & Co. KG, Südlohn-Oeding, Germany) (Table 2). They received 7.5 g CLA/day (50% *c*9,*t*11- and 50% *t*10,*c*12-CLA) starting two weeks before expected calving and followed by 20 g CLA/d (50% *c*9,*t*11- and 50% *t*10,*c*12-CLA) during the first 28 days of lactation. CLA supplement was offered at the bunk once daily immediately after milking (0600 h) while cows were fixed in the feed fence. Refusals of CLA were weighed and recorded.

**Table 1:** Ingredients and chemical composition of lactational and basal diet^1^.

Variable	Lactational diet	Basal diet
Ingredient, %		
Corn silage	60	43
Grass silage	23	25
Hay	4	32
Concentrates^2^	12	-
Mineral mix^3^	1	-
		
Chemical analysis, %		
DM	45.2	52.0
CP	12.2	12.9
CF	18.6	19.2
NFC	26.4	9.92
Ether extract	3.16	3.16
		
NE_L_, Mcal/kg	1.55	1.36

^1^Lactational diet was fed to all cows postpartum and basal diet was fed to all cows prepartum.

^2 ^Composition: corn gluten 18.4%, turnips molasses chips 13.8%, wheat 10.0%, triticale 10.0%, rape cake 10.0%, maize 8.8%, malt germ 6.0%, grain distillation residual (ProtiGrain) 5%, rape extraction grist 5%, rumen protected rape extraction grist 5%, palm corn cake 3.3%, soy extraction grist 2.8%, sodium bicarbonate 1.0%, calcium bicarbonate 0.99%, plant oil (palm coconut) 0.40%.

^3 ^The mix contained calcium 14%, sodium 10%, phosphorous 5%, magnesium 5%.

**Table 2:** Ingredients of CLA supplement^1^.

Variable	
Ingredient, %	
Soybean	52
Glucose	10
CLA	15
*There of c9, t11-CLA*	7.5
*t10, c12-CLA*	7.5
Biscuit flour	4
Wheat bran	4
Cornflakes	3.5
Magnesium phosphate	3.5
Malt sprouts	2.5
Brewer's yeast	1.5
lactalbumin powder	0.8
Soybean oil	0.2
	
NE_L_, Mcal/kg	3.35

^1 ^Cows received 7.5 g CLA/day (50% *c*9,*t*11- and 50% *t*10,*c*12-CLA) starting two weeks before expected calving and followed by 20 g CLA/day (50% *c*9,*t*11- and 50% *t*10,*c*12-CLA) during the first 28 days of lactation

After parturition, cows were milked twice daily (0415 and 1545 h) and milk yields were recorded at each milking until day 56. Milk samples were taken at the evening milking. Samples were separated during milking into sample vessels (capacity about 1 liter) controlled by milk flow rate and total amount of milk (Metatron P21, GEA WestfaliaSurge, Boenen, Germany). One aliquot was stored at 4°C for a maximum of 10 days with a preservative (acidiol) until analyses of milk composition. A second aliquot was stored at -20°C until analyzed for fatty acid composition. Milk composition was analyzed daily during the first week of lactation and thereafter twice a week for 7 weeks (from week 2 to week 8 postpartum). Fatty acid (FA) composition was examined twice a week during the first 4 weeks postpartum and weekly during the following 4 weeks (from week 5 to week 8 postpartum).

Jugular blood samples were collected in the morning (0700 h) at calving and at weeks 1, 2, 4, 6, and 8 of lactation. Blood serum was harvested following centrifugation (2000 × *g*, 15 min at 4°C) and stored in three aliquots at -20°C until analyzed for total bilirubin (TB), glucose, non-esterified fatty acids (NEFA) and betahydroxybutyrate (BHB). Liver biopsies were obtained (Bard^® ^MAGNUM™, Covington, USA) in the morning (0800 h) at weeks 1, 2, 4 and 8 of lactation. Liver tissue (approx. 100 mg) was frozen immediately in liquid nitrogen, and stored at -80°C until analyzing for gene expression.

### Milk composition analysis

Milk protein, fat, lactose, urea and pH were analyzed by infrared-spectrophotometric technique (MilkoScan™ FT6000) and somatic cell count was determined by fluorescence-optical counting system (Fossomatic™ FC) in the laboratories of Milchprüfring Bayern e.V., Germany.

### Milk fatty acid analysis

The FA composition of milk samples was determined using FA methyl esters (FAME) prepared by transesterification with TSMH (trimethylsulfonium hydroxide) at room temperature. FAMEs were analyzed using gas chromatography (GC 6890, AgilentTechnologies, Waldbronn, Germany) to determine isomer distribution patterns. FA were quantified by use of Chromeleon^® ^6.8 Chromatography Software (Dionex, USA).

### Blood serum analysis

Glucose, NEFA, BHB, and TB were analyzed with an automated clinical chemistry analyzer (ABX Pentra 400, Horiba, Montpellier, France). The hexokinase method was applied for glucose analysis and NEFA concentrations were determined with the enzymatic reactions (both Hoffmann La-Roche, Basel, Switzerland). BHB measurement was performed by using an enzymatic analysis (Sigma-Aldrich Diagnostics, Munich, Germany). TB was analyzed via Jendrassik/Grof reaction [4]. The clinical chemistry analyzer was calibrated and controls assayed daily according to the manufacturer's instructions to ensure acceptable assay performance.

### Gene expression

Total RNA was isolated from liver tissue samples according to the manufacturer's instructions of peqGOLD TriFast™. RNA was quantified by spectrophotometry (BioPhotometer, Eppendorf, Hamburg) and diluted in RNase-free water. Degradation of the RNA was measured with the Agilent 2100 Bioanalyzer (Agilent Technologies, Waldbronn, Germany) in connection with the RNA 6000 Nano Assay. Gene expression was measured by reverse transcription quantitative polymerase chain reaction (RT-qPCR) (SuperScript™ III Platinum^® ^SYBR^® ^Green One PCR Kit, Invitrogen, Karlsruhe, Germany) using the RotorGene3000 (Corbett Research, Cambridge, United Kingdom). Primer for proliferator-activated receptor-alpha (PPARα), PPARγ, sterol regulatory element-binding protein-1 (SREBP1) and tumor necrosis factor-alpha (TNFα) were designed using Primer3 online-software and synthesized by Metabion International AG (Martinsried, Germany, Table 3). The mean of the two housekeeping genes, histone and ubiquitin, was calculated for the reference index and used for normalization. Δ quantitative Cycle (Cq)-values were calculated as ΔCq = Cq_target gene _- meanCq_refence genes _and ΔΔCq-values were calculated according to ΔΔCq = ΔCq_target gene _- meanΔCq_refence genes_.

**Table 3:** Sequences of PCR primers^1^.

Primer		Sequence (5' → 3')
Histone	forward	ACT TGC TAC AAA AGC CGC TC
	reverse	ACT TGC CTC CTG CAA AGC AC
Ubiquitin	forward	AGA TCC AGG ATA AGG AAG GCA T
	reverse	GCT CCA CCT CCA GGG TGA T
PPARα	forward	GGA TGT CCC ATA ACG CGA TTC G
	reverse	TCG TGG ATG ACG AAA GGC GG
PPARγ	forward	CTC CAA GAG TAC CAA AGT GCA ATC
	reverse	CCG GAA GAA ACC CTT GCA TC
SREBP1	forward	CCA GCT GAC AGC TCC ATT GA
	reverse	TGC GCG CCA CAA GGA
TNFα	forward	CCA CGT TGT AGC CGA CAT C
	reverse	CCC TGA AGA GGA CCT GTG AG

^1 ^Primers were designed using Primer3 online software and synthesized by metabion international AG (Martinsried, Germany).

### Statistical analysis

Endpoints measured repeatedly (milk yield, milk composition and milk FA profile) were reduced to weekly means before statistical analysis. Differences among treatments (group) and comparisons between times (week) were analyzed by repeated measures ANOVA using Bonferroni's t-test (Sigma-Stat v.3.00 and the PASW Statistics 17, both SPSS Inc., Chicago, USA).

The effects of group and week were considered as fixed effects with week of experiment as a repeated measurement and with cow within dietary treatment (group) as the subject. Orthogonal polynomial contrast was used to describe linear, quadratic or cubic trends over time (week by group interaction) and group effects. All data are presented as mean ± standard deviation (SD). Means were considered to differ significantly in case *P *< 0.05.

## Results

The CLA supplement was insufficiently accepted by the animals after parturition: on average, only 61.5% of the intended amount of 20 g CLA per day (10 g *c*9,*t*11- and 10 g *t*10,*c*12-CLA) was ingested. In the first week postpartum cows ingested 10.1 ± 7.8 g CLA per day, 11.5 ± 7.7 g CLA at week 2 postpartum, 15.2 ± 6.4 CLA at week 3 postpartum and 12.4 ± 6.5 g CLA at week 4 of lactation (50% *c*9,*t*11 and 50% *t*10,*c*12-CLA). On average, cows ingested 12.3 ± 4.7 g CLA/day during the treatment period. Results were calculated for two timeframes: from day 1 postpartum until day 28 postpartum (CLA supplemented period) and from day 29 postpartum until day 56 postpartum. Milk yield, milk protein, milk fat, and urea content did not differ between the two groups (Table 4).

**Table 4:** Means ± SD for milk yield and milk composition during and after the treatment period^1^.

	Control (n = 5)				CLA (n = 5)			
	1 - 28 DIM		29 - 56 DIM		1 - 28 DIM		29 - 56 DIM	
Variable	mean^5^	SD	mean	SD	mean	SD	mean	SD
milk yield^2^, kg/day	24.5	2.8	28.8	2.3	24.5	3.3	28.9	2.1
3.5% FCM^3^, kg/day	30.9	5.4	34.7	5.7	30.4	2.6	33.0	3.5
milk fat^4^, %	6.10	0.99	5.27	0.78	5.77	0.14	4.97	0.30
milk fat^4^, kg/day	1.49	0.15	1.52	0.12	1.41	0.16	1.44	0.12
milk protein^4^, %	3.81	0.12	3.31	0.13	3.82	0.15	3.42	0.13
milk protein^4^, kg/day	0.93	0.09	0.95	0.07	0.94	0.09	0.99	0.08
milk lactose^4^, %	4.75	0.28	5.02	0.03	4.72	0.25	4.94	0.02
milk lactose^4^, kg/day	1.17	0.25	1.43	0.03	1.14	0.28	1.42	0.02
urea^4^, mmol/L	6.25	1.14	5.28	1.27	5.63	1.26	5.38	0.96

^1^CLA supplemented timeframe: d 1 till d 28 of lactation; five primiparous cows received a special supplement including rumen-protected CLA (10 g *c*9,*t*11-CLA/day and *t*10,*c*12-CLA/day).

^2^Milk yield was recorded at each milking.

^3^FCM was calculated like following: (fat [%] × 0.15 + 0.4) × milk yield [kg/day]

^4^Milk composition was analyzed once daily in the first week after parturition and twice a week from week 2 until week 8 postpartum.

^5^Milk yield and milk composition values were reduced to weekly means, means from wk 1 till 4 are pooled to timeframe 1 - 28 DIM, means from week 5 till 8 are pooled to timeframe 29 - 56 DIM

Metabolic key parameters did not differ between groups during treatment and from week 5 to week 8 (Table 5).

**Table 5:** Means ± SD for blood serum metabolites^1 ^during and after the treatment period^2^.

	Control (n = 5)				CLA (n = 5)			
	1 - 28 DIM		29 - 56 DIM		1 - 28 DIM		29 - 56 DIM	
Variable	mean	SD	mean	SD	mean	SD	mean	SD
TB^3^, μmol/L	7.01	4.52	3.58	0.74	4.51	0.73	3.10	0.70
Glucose, mmol/L	3.40	0.11	3.53	0.06	3.47	0.28	3.53	0.19
NEFA^4^, μmol/L	483	174	165	105	378	119	152	61.1
BHB^5^, mmol/L	0.54	0.14	0.22	0.08	0.49	0.28	0.39	0.04

^1^Jugular blood samples were collected at week 0, 1, 2, 4, 6, 8. Values from week 0, 1, 2, 4 were calculated for 1 - 28 DIM, values from week 6 and 8 were calculated for 29 - 56 DIM.

^2^CLA supplemented timeframe: d 1 until d 28 of lactation; five primiparous cows received a special supplement including rumen-protected CLA (10 g *c*9,*t*11-CLA/day and *t*10,*c*12-CLA/day).

^3^total bilirubin.

^4^non-esterified fatty acids.

^5^betahydroxybutyrate.

Absorbed CLA was detectable in milk fat during the supplementation timeframe, resulting in a shift in the FA composition of milk fat (Table 6). On average over the entire treatment period, there was a reduction in the yield of saturated fatty acids in the milk fat of cows receiving CLA, together with a higher content of monounsaturated and *trans *FA. Contents of *c*9,*t*11-CLA were higher in milk fat of CLA supplemented cows compared to the control group (0.73 ± 0.04 g/100 g fat *vs*. 0.64 ± 0.01 g/100 g fat). In addition, contents of *t*10,*c*12-CLA in milk fat of CLA supplemented cows were significantly higher compared to the control group (0.02 ± 0.01 g/100 g fat *vs*. 0.00 ± 0.00 g/100 g fat, *P *= 0.002).

**Table 6:** Means ± SD for fatty acid composition of milk fat from cows received *c*9,*t*11- and *t*10,*c*12-CLA and for the control group, during treatment period^1^ and post-treatment.

	Control (N = 5)				CLA (N = 5)				*P*-value	*P*-value	*P*-value	*P*-value
	1 - 28 DIM		29 - 56 DIM		1 - 28 DIM		29 - 56 DIM		Control	CLA	Control vs. CLA	Control vs. CLA
	mean	SD	mean	SD	mean	SD	mean	SD	1-28 vs. 29-56	1-28 vs. 29-56	1-28	29-56
Fatty acid, g/100 g fat												
4:0	1.40	0.10	1.20	0.12	1.38	0.11	1.24	0.04				
6:0	1.14	0.06	1.18	0.06	1.13	0.13	1.23	0.02				
8:0	0.84	0.03	0.94	0.02	0.86	0.10	1.01	0.04	0.003			0.038
10:0	1.91	0.12	2.39	0.20	1.95	0.20	2.55	0.13	0.011	0.012		
10:1	0.15	0.03	0.25	0.02	0.15	0.02	0.28	0.03	0.09	0.003		
11:0	0.05	0.02	0.08	0.08	0.04	0.01	0.07	0.01		0.013		
12:0	2.90	0.18	3.79	0.44	2.87	0.41	4.04	0.30	0.013	0.016		
12:1 *cis*-9	0.04	0.01	0.07	0.01	0.03	0.01	0.08	0.01	0.007	0.002		
iso-13:0	0.04	0.01	0.08	0.01	0.04	0.01	0.09	0.02	0.008	0.03		
13:0	0.09	0.02	0.14	0.09	0.07	0.01	0.12	0.02		0.013		
iso-14:0	0.09	0.03	0.11	0.04	0.08	0.00	0.10	0.02				
14:0	9.14	0.42	11.35	0.45	8.84	0.39	11.27	0.37	0.001	0.001		
14:1 *cis*-9	0.57	0.08	0.86	0.13	0.57	0.05	0.88	0.13	0.013	0.0021		
iso-15:0	0.10	0.00	0.11	0.00	0.09	0.01	0.10	0.01	0.012			
anteiso-15:0	0.27	0.04	0.36	0.04	0.26	0.03	0.36	0.04	0.034	0.023		
15:0	0.85	0.10	1.15	0.48	0.70	0.09	1.01	0.07		0.01		
iso-16:0	0.23	0.03	0.23	0.07	0.21	0.02	0.22	0.01				
16:0	26.17	0.82	27.90	0.95	25.74	1.57	26.62	0.95	0.05			
16:1 *trans*-9	0.17	0.00	0.17	0.01	0.17	0.01	0.16	0.01			0.04	
16:1 *cis*-9	1.48	0.04	1.40	0.06	1.65	0.12	1.38	0.13				
iso-17:0	0.36	0.02	0.42	0.04	0.36	0.04	0.43	0.03				
anteiso-17:0	0.43	0.04	0.45	0.04	0.42	0.05	0.44	0.04				
17:0	0.71	0.05	0.63	0.11	0.64	0.06	0.58	0.01				
17:1 *cis*-9	0.32	0.02	0.27	0.02	0.34	0.04	0.24	0.02	0.014	0.016		
iso-18:0	0.09	0.01	0.07	0.01	0.09	0.01	0.06	0.00		0.011		
18:0	11.84	0.44	9.52	0.46	11.59	0.54	10.13	0.29	0.001	0.014		
18:1 *trans*-9	0.59	0.12	0.61	0.03	0.63	0.03	0.66	0.05				
18:1 *trans*-10	0.30	0.02	0.36	0.05	0.31	0.05	0.39	0.05				
18:1 *trans*-11	1.48	0.43	1.29	0.13	1.49	0.11	1.29	0.08				
18:1 *cis*-9	25.78	1.26	22.19	0.94	26.45	1.48	22.44	0.17	0.009	0.01		
18:1 *cis*-11	1.09	0.06	1.01	0.17	1.10	0.11	1.00	0.04				
18:1 *cis*-12	0.24	0.04	0.27	0.02	0.27	0.05	0.28	0.02				
18:1 *cis*-13	0.11	0.02	0.07	0.01	0.11	0.05	0.08	0.03	0.03			
18:2 *trans*-9, *trans*-12	1.83	0.08	1.82	0.10	2.11	0.20	1.97	0.13			0.048	
18:2 *cis*-9, *trans*-11	0.64	0.10	0.69	0.05	0.73	0.04	0.68	0.07				
18:2 *trans*-10, *cis*-12	0.00	0.00	0.00	0.00	0.02	0.01	0.00	0.00		0.008	0.002	
18:2 *trans*-9, *trans*-11	0.02	0.01	0.01	0.00	0.03	0.00	0.01	0.00		0.002		
18:3 *cis*-9, *cis*-12, *cis*-15	0.43	0.03	0.40	0.06	0.43	0.01	0.36	0.01		0.003		
19:0	0.06	0.00	0.05	0.00	0.04	0.01	0.05	0.01			0.015	
20:0	0.14	0.01	0.13	0.00	0.13	0.00	0.14	0.00		0.004	0.029	0.027
20:1 *cis*-11	0.11	0.00	0.11	0.02	0.10	0.01	0.10	0.02				
20:2 *cis*-11, *cis*-14	0.03	0.01	0.03	0.01	0.05	0.01	0.05	0.03				
21:0	0.02	0.00	0.02	0.00	0.01	0.00	0.02	0.00		0.026	0.004	
20:3 *cis*-8, *cis*-11, *cis*-14	0.08	0.01	0.11	0.01	0.10	0.01	0.13	0.00	0.014	0.008		
20:4 *cis*-5, *cis*-8, *cis*-11, *cis*-14	0.13	0.03	0.12	0.01	0.11	0.01	0.10	0.02				
22:0	0.03	0.01	0.03	0.00	0.02	0.00	0.03	0.00		0.01		
20:5 *cis*-5. *cis*-8, *cis*-11, *cis*-14, *cis*-17	0.03	0.01	0.02	0.00	0.02	0.00	0.02	0.00				0.036
24:0	0.03	0.01	0.02	0.00	0.01	0.00	0.02	0.01			0.005	
22:5 *cis*-7, *cis*-10, *cis*-13, *cis*-16, *cis*-19	0.12	0.02	0.10	0.01	0.09	0.01	0.08	0.01				
Other	5.36		5.43		5.35		5.44					
												
Summation, g/100 g fat												
SFA^2^	58.9	0.87	62.3	1.01	57.5	1.95	61.9	0.07	0.005	0.018		
MUFA^3^	29.9	1.23	26.5	0.87	30.8	1.67	26.7	0.27	0.01	0.014		
PUFA^4^	0.39	0.06	0.38	0.03	0.39	0.01	0.38	0.03				
*t*FA^5^	4.37	0.58	4.25	0.17	4.71	0.34	4.46	0.21				
CLA^6^	1.12	0.07	1.14	0.10	1.20	0.05	1.08	0.06		0.046		

^1^CLA supplemented timeframe: d 1 till d 28 of lactation; five primiparous cows received a special supplement including rumen-protected CLA (10 g *c*9,*t*11-CLA/day and *t*10,*c*12-CLA/day). Post-treatment timeframe: 29 - 56 DIM.

^2^saturated fatty acids

^3^monounsaturated fatty acids

^4^polyunsaturated fatty acids

^5^*trans *fatty acids

^6^conjugated linoleic acids

Total RNA quantity and RNA integrity number (RIN) values were similar for cows in the control group (RNA concentrations: 1276 ± 1016 ng/μl; RIN values: 6.5 ± 2.3) and for cows in CLA supplemented group (RNA concentrations = 1109 ± 986 ng/μl; RIN values: 6.6 ± 1.9). mRNA levels of histone and ubiquitin were tested for normal distribution. Constant mRNA levels of histone and ubiquitin was manifested by analysis of variance. ΔCq-values and ΔΔCq-values of the genes PPARα, PPARγ, SREBP1 and TNFα did not differ between the two groups and over the weeks (Table 7).

**Table 7:** ΔCq^1^-values (mean ± SD) of hepatic mRNA expression of the genes PPARα^2^, PPARγ^3^, SREBP1^4^, TNFα^5^in CLA supplemented cows vs. control group before (week -1), during (week 2 and 4) and after (week 8) treatment^6^.

	Control (n = 5)								CLA (n = 5)							
	week -1		week 2		week 4		week 8		week -1		week 2		week 4		week 8	
	mean	SD	mean	SD	mean	SD	mean	SD	mean	SD	mean	SD	mean	SD	mean	SD
ΔPPARα	1.5	0.3	1.4	0.2	1.9	0.3	1.7	0.5	2.0	0.1	1.4	0.3	1.9	0.7	2.0	0.5
ΔPPARγ	7.8	0.8	9.6	2.3	9.8	2.4	9.4	0.8	9.1	0.3	8.6	0.2	9.2	0.7	9.2	0.2
ΔSREBP1	2.3	1.6	4.0	2.9	4.0	1.8	4.2	1.6	3.0	0.2	3.7	0.7	4.1	1.2	3.1	0.5
ΔTNFα	9.3	0.6	8.9	0.4	9.4	0.7	9.4	1.2	8.5	0.4	8.0	1.1	8.5	1.5	8.4	0.4

^1 ^ΔCq-values were calculated as ΔCq = Cq_target gene _- meanCq_refence genes_

^2 ^peroxisome proliferator-activated receptor-alpha.

^3^peroxisome proliferator-activated receptor-gamma.

^4^sterol regulatory element-binding protein-1.

^5^tumor necrosis factor-alpha.

^6^CLA supplemented timeframe: day 1 till day 28 of lactation; five primiparous cows received a special supplement including rumen-protected CLA (10 g *c*9,*t*11-CLA/day and *t*10,*c*12-CLA/day).

## Discussion

The transition period between late pregnancy and early lactation is characterized by a shift in nutrient partitioning that requires extensive coordination of metabolism to ensure an adequate supply of nutrients to support milk synthesis [5]. Due to this metabolic adaptation, the CLA supplementation in this project was designed to start before parturition and to take place along the whole transition period.

In the present study, CLA supplementation did not affect milk fat content. This differs from other studies conducted with cows during established lactation, in which feeding rumen-protected CLA [6-9] or abomasal infusion of CLA [10-13] or intravenous infusions of CLA [14] resulted in a reduction of milk fat content. An explanation for the lack of a CLA response in milk fat during the first few weeks postpartum is unknown [8]. Our analysis indicates that *c*9,*t*11- and *t*10,*c*12-CLA were consistently transferred to milk fat throughout the treatment period. Contents of *c*9,*t*11- and *t*10,*c*12-CLA in milk of CLA supplemented cows were significantly higher compared to the *c*9,*t*11- and *t*10,*c*12-CLA content in milk of cows of the control group.

Peterson *et al*. [3] found that *t*10,*c*12-CLA was also transferred to milk fat and that the milk fat content of *t*10,*c*12-CLA are curvilinearly related to reduced milk fat yield, according to de Veth *et al*. [15]. However in our study we could not demonstrate such a decrease of milk fat yield. It can be speculated that at the onset of lactation the essential cellular signaling systems are attenuated such that *t*10,*c*12-CLA is unable to elicit the coordinated reduction in the expression of genes for key lipogenic enzymes.

Furthermore in milk of CLA supplemented cows, contents of short chain (< C 10) and middle chain FA (C 10 - C 16) were reduced and the amount of long chain FA (> C 16) was increased. These results go in line with previous results from studies conducted during early and mid lactation [8,9,16,17].

Results from the present study with transition cows are partially similar to those observed after administration of CLA to cows in established lactation. In established lactation milk yield and milk protein content were relatively unaffected by abomasal administration of CLA or CLA feeding [3,11,13]. In our study CLA supplementation did not affect milk yield and milk protein content.

Bernal-Santos *et al*. [8] presented the first study in which rumen-protected CLA was fed during the prepartum period. Supplementation had no effect on measured performance variables and plasma metabolites. In our study, CLA supplementation had no effects on concentrations of TB, glucose, NEFA and BHB in blood serum. Because of sustained lipomobilization the metabolic situation could not be improved by feeding CLA. Comparable results were obtained by Perfield *et al*. [7]. In addition, CLA supplementation had no effects on hepatic mRNA levels of PPARα, PPARγ, SREBP1 and TNFα. Comparable studies, in which effects of CLA supplementation on hepatic gene expression in dairy cows were measured, do not exist to our knowledge. Previous studies in rodents revealed effects of *c*9,*t*11- and *t*10,*c*12-CLA on gene expression of fatty acid synthesis, fatty acid oxidation and drug detoxification-associated enzymes in liver tissue [18-20].

Our results go in line with previous results stating that *t*10,*c*12-CLA affects primarily the *de novo *synthesis of FA in the alveolar epithelial cells of the mammary gland but does not inhibit peripheral lipomobilization. The transition period is associated with an increased mobilization of body fat reserves, which results in an increased mammary uptake of circulating NEFA and their use to synthesize milk fat triglycerides [13]. This is one explanation of the considerably higher fat percentage of bovine milk in the first weeks of lactation when precursors for milk synthesis are not completely available from feed [1].

Obviously, during the first weeks of lactation, the milk fat depressing effects of *t*10,*c*12-CLA intake are less pronounced compared to mid or late lactation. This may be explained by a low contribution of long-chain FAs originating from lipomobilization to milk fat in mid lactation while the proportion of *t*10,*c*12-CLA-dependend *de novo *synthesized FAs is high compared to the first weeks of lactation. The study did not provide evidence that CLA affected substrate partitioning in the body of the cows which may be explained by the comparatively small amounts fed compared to rodent studies.

The present study is the first describing supplemention of primiparous cows with CLA during the first four weeks of lactation. However, due to the limited number of animals per treatment group, the small amount of supplemented CLA and the restricted supplementation period during early lactation, it is difficult to make any definitive conclusions about the metabolic benefits of CLA supplementation.

## Conclusions

Supplementation of *c*9,*t*11- and *t*10,*c*12-CLA during the first four weeks of lactation resulted in an increase of these specific CLA isomers provided during treatment. Therefore all the CLA isomers were taken up by the mammary gland and incorporated into milk fat. During the first four weeks of lactation, however, CLA supplementation did not affect milk yield, milk composition, blood serum metabolites and gene expression in liver of primiparous cows.

## Competing interests

The authors declare that they have no competing interests.

## Authors' contributions

TS was responsible for the CLA feeding as well as for all sample obtention, fatty acid composition analysis, mRNA extraction from liver tissue, RT-qPCR performance, and statistical analysis of the results. GS assisted in blood sampling. HK briefed TS in working with the HPLC. MK created the experimental design and supervised the study. SW performed liver biopsies. HHDM was the project leader and supervised the study. All authors read and approved the final manuscript.
