# Rumen-protected conjugated linoleic acid supplementation to dairy cows in late pregnancy and early lactation: effects on milk composition, milk yield, blood metabolites and gene expression in liver

**DOI:** 10.1186/1751-0147-52-32

**Published:** 2010-05-20

**Authors:** Tanja Sigl, Gregor Schlamberger, Hermine Kienberger, Steffi Wiedemann, Heinrich HD Meyer, Martin Kaske

**Affiliations:** 1 Physiology Weihenstephan, Technische Universitaet Muenchen, Weihenstephaner Berg 3, Freising-Weihenstephan, Germany; 2 Bioanalytik, Technische Universitaet Muenchen, Versuchsstation Thalhausen, Freising-Weihenstephan, Germany; 3 Clinic for Cattle, University of Veterinary Medicine, Bischofsholer Damm 15, Hannover, Germany

## Correction

After publication of this work [1] we realised that some of the values were incorrect in Table 2 Additional file 1. We have corrected these in the updated table.

## Supplementary Material

Additional file 1 Corrected table 2. Click here for file
