# Supplementary material for: Rumen-protected conjugated linoleic acid supplementation to dairy cows in late pregnancy and early lactation: effects on milk composition, milk yield, blood metabolites and gene expression in liver
Source: Acta Vet Scand. 2010 May 20;52(1):32. doi: 10.1186/1751-0147-52-32 (PMC2880086; doi:10.1186/1751-0147-52-32)
Supplement: Additional file 1 — Corrected table 2. [file 1751-0147-52-32-S1.DOCX]

**Table 2.** Ingredients of CLA supplement^1^.

| **Variable** |  |
| --- | --- |
| Ingredient, % |  |
| Soybean | 52 |
| Glucose | 10 |
| Palm fat | 15 |
| *There of c9,t11-CLA* | 0.75 |
| *t10,c12-CLA* | 0.75 |
| Biscuit flour | 4 |
| Wheat bran | 4 |
| Cornflakes | 3.5 |
| Magnesium phosphate | 3.5 |
| Malt sprouts | 2.5 |
| Brewer`s yeast | 1.5 |
| lactalbumin powder | 0.8 |
| Soybean oil | 0.2 |
|  |  |
| NE_L_, Mcal/kg | 3.35 |

^1^ Cows received 7.5 g CLA/day (50% *c*9,*t*11- and 50% *t*10,*c*12-CLA) starting two weeks before expected calving and followed by 20 g CLA/day (50% *c*9,*t*11- and 50% *t*10,*c*12-CLA) during the first 28 days of lactation
